# Frequency Following Response (FFR) in children with developmental language disorder

**DOI:** 10.1016/j.bjorl.2026.101939

**Published:** 2026-10-31

**Authors:** Gustavo Fernando Tognini Rodrigues, Fabiana Cristina Mendonça de Araújo, Doris Ruthy Lewis

**Affiliations:** a Pontifícia Universidade Católica de São Paulo (PUC-SP), São Paulo, SP, Brazil; b Universidade Federal do Rio Grande do Norte (UFRN), Rio Grande do Norte, RN, Brazil

**Keywords:** Auditory brainstem response, Child, Developmental language disorder, Frequency following response, Speech perception

## Abstract

**Objective:**

To study the frequency following response in children with developmental language disorder.

**Methods:**

An exploratory pilot, observational, cross-sectional, and prospective study was conducted with children aged four to eleven years, divided into a Developmental Language Disorder group and a typical development control group, focusing on the feasibility of FFR acquisition and time- and frequency-domain analysis. The Frequency Following Response was elicited using the synthesized syllable /da/ at eighty decibels sound pressure level. The responses were analyzed in both time and frequency domains. In the time domain, the amplitude of the waves V and A complex and absolute latencies of waves V, A, C, D, E, F, and O were evaluated. Crucially, frequency-domain analysis was performed using MATLAB software, where the Fast Fourier Transform was applied to the sustained portion of the response to quantify the amplitudes of the fundamental frequency and the first two formants.

**Results:**

The time-domain analysis revealed no statistically significant differences in the mean latencies of the waves or the amplitude between the groups. In the frequency-domain analysis using MATLAB, the comparison of amplitudes for the fundamental frequency and the first formants also showed no significant differences between the Developmental Language Disorder and control groups. However, the large wave summation averages demonstrated wave latencies, fundamental frequency, and first formants consistent with the literature, confirming the reproducibility of the test.

**Conclusion:**

The Frequency Following Response is a viable auditory evoked potential in clinical settings for evaluating speech-sound coding in the auditory brainstem. Although no statistical significance was found between the groups, likely due to sample size and clinical heterogeneity, the method proved reliable, and frequency-domain analysis using MATLAB provided a robust objective assessment of temporal processing.

## Highlights

• FFR objectively assesses auditory processing in children with DLD.
• FFR measures brainstem neural responses to speech sounds.
• FFR may contribute to the investigation of auditory processing in children with DLD.
• FFR evaluates speech sound processing in the auditory brainstem.

## Introduction

The integrity of the peripheral and central auditory systems is essential for oral language acquisition. Understanding acoustic elements and their phonetic representations as language formants is one of the means of speech development. The Frequency Following Response (FFR) to speech stimuli can be used to understand how the auditory pathway processes verbal sounds, allowing observation of the acoustic properties of speech formants, which appear preserved in brainstem responses.

Complex-sounds Auditory Brainstem Response (cABR), or Frequency Following Response- FFR, allows the assessment of how behavioral sounds such as speech and music are processed in the brain. They are subcortical potentials that preserve the temporal and spectral characteristics of the sound and make it possible to view difficulties and specific improvements in auditory processing.^1^, ^2^, ^3^, ^4^, ^5^, ^6^, ^7^, ^8^

The FFR results from the brainstem’s neural response to the timing of a sound's brief (transient) and ongoing (sustained) components. This response appears as repeated neural discharge patterns occurring at consistent time intervals, mirroring sound timing. Synchronizing these neural discharges to sound timing is called phase locking.^1^^,^^9^, ^10^, ^11^

The complex sound stimulus elicits seven sequential negative-peak responses, named V, A, C, D, E, F, and O. The VA Complex represents the stimulus's transient portion. Peaks D to F express the sustained portion of the stimulus and are considered the electrophysiological representation of the FFR that contains the fundamental frequency of the stimulus. The C and O peaks are also considered transient because they correspond to the start and end of the sustained stimulus, respectively.^1^^,^^9^, ^10^, ^11^

Developmental Language Disorder (DLD) is highly heterogeneous. It can affect language production and/or comprehension, with varying degrees of severity across different aspects of language processing (e.g., lexical, morphosyntactic, semantic, and pragmatic). It is among the most common developmental disorders, with neuropsychological sequelae in about 40%–50% of cases.^12^

Children with DLD struggle to identify tones with short interstimulus intervals or short verbal stimuli, and to discriminate consonant-vowel syllables with shorter formant transition durations, showing deficits in temporal resolution.^13^, ^14^, ^15^, ^16^

The FFR directly measures speech sounds' temporal conduction in the auditory brainstem, helping clarify temporal processing difficulties in individuals with Developmental Language Disorder (DLD).

The aim of this study was to investigate Frequency Following Response (FFR) characteristics in children with Developmental Language Disorder compared to children with typical language development.

## Methods

The project was submitted to the Research Ethics Committee of Pontifical Catholic University of São Paulo (PUC-SP) and approved under the CAAE number 55381821.2.0000.5482. Those responsible for the children signed the informed consent (free and informed consent term – TCLE). This was designed as an exploratory pilot, observational, cross-sectional, and prospective study, focused on assessing the feasibility of FFR acquisition and time- and frequency-domain analysis in children with Developmental Language Disorder compared with typically developing children.

The study was carried out at Division of Education and Rehabilitation of Communication Disorders (DERDIC), a reference service for hearing and language services accredited by the Ministry of Health.

The exams were performed at the Centro Audição na Criança (CeAC) of Pontifical Catholic University of São Paulo (PUC-SP). CeAC is the research laboratory for the Children's Hearing research group in the Post-Graduation Program in Human Communication and Health at PUC-SP.

Subject: Nine children were invited to participate in the study, aged between four and eleven years, with a diagnosis of Development Language Disorder (DLD), between March and June of 2022.

These children were diagnosed with DLD, considering severe and persistent difficulties with understanding and/or expressing language that affect daily life and academic performances, with no other medical condition present; linguistic expression worse than comprehension and alteration in two or more language subsystems.^17^^,^^18^ The most comprehensive language test available in Brazil is the ABFW Child Language Test.^19^ All children in this group had ABFW scores at least 1.25 standard deviations below the average for vocabulary and phonology.^20^

Children with DLD were recruited from the Phoniatrics outpatient clinic at DERDIC-PUC-SP and a private Otorhinolaryngology/Phoniatrics clinic, where they had received over 12-months of follow-up and speech-language therapy. The nine control-group children, all with typical neurological development and normal hearing, had no history of recurrent otitis or school difficulties. They underwent the same hearing evaluation as the DLD group, including a neurological exam by a Pediatric Neurologist to confirm typical development.

Inclusion criteria: normal hearing, confirmed by means of audiological evaluation with tonal audiometry, at least in the frequencies of 500, 1000, 2000, and 4000 Hz, with headphones and conditioning, if necessary. Acoustic immittance test (tympanometry) and recording of normal Transient Evoked Otoacoustic Emissions (TEOAEs) and Distortion Product (DPOAEs), on the same day as the FFR. ABR click of 100 microseconds, with alternating polarity, stimulus presentation rate of 21.1 stimuli per second, presentation window of 12-milliseconds, intensity of 80 dB SPL, alternating polarity, high pass filters of 30 Hz and low pass of 1500 Hz, analyzed waves I, III, and V in absolute latencies, latency of interpeak intervals I‒III, III‒V, and IV. The values of absolute latencies and interpeak intervals will be compared with the literature on standardized normality from the British Society of Audiology, published in September 2019.

Exclusion Criteria: Children diagnosed with neurodevelopmental disorders, syndromes, hearing loss, ear malformations, recurrent otitis, genetic or syndromic language disorders, or chronic childhood encephalopathy.

### Data collection procedure

Electrodes were positioned vertically for both click ABR and cABR with complex-sound stimuli: active at the earlobe or mastoid ipsilateral to the stimulus, reference (A1) at midline Cz (10–20 system), and ground on the forehead.

The FFR using the syllable /da/ was analyzed in a static window, considering its transient and sustained portions, across both the Time domain and the Frequency domain.

The sound stimulus, syllable /da/, was presented at a fundamental Frequency (F0) ≤300 Hz to enable robust FFR responses without losing specificity, at 80 dB SPL in the right ear; the left ear was tested when possible. The synthesized /da/ stimulus duration in the STM file (SmartEP device, IHS v5.36) was 40 ms. Two sub-averages of 3,000 sweeps were combined to produce a 6,000-sweep trace for wave identification. The stimulus rate was 10.9 per second, with a 15 ms pre-stimulus window extending to 57 ms (−15 ms to 57 ms). Artifacts >35 microvolts were rejected. The stimulus had alternating polarity. Filters were a 100 Hz high-pass and a 1,500 Hz low-pass.^21^

### Analysis

1 For FFR analysis, the transient and sustained portions were considered. In the evaluation of the transient portion, the amplitude of the VA complex and the latency of waves V, A, and C.^1^^,^^22^
2 In the evaluation of the sustained portion, in addition to the latency of the D, E, and F waves, the Signal-to-Noise Ratio (SNR) and RMS were analyzed (in the time domain), and (in the frequency domain), the Fast Fourier Transform (FFT). For RMS and FFT analysis, MATLAB engineering software, version 2015, was used after exporting the grand average of 6,000 scans (the sum of two records of 3,000 FFR scans) from IHS SmartEP (in an ASCII file) into the software environment.^22^
3 The FFR was analyzed at two different times. Initially, through summation tracing in the equipment IHS- SmartEp device, and later in MATLAB software. From this initial assessment, descriptive and comparative analyses were performed on the absolute latencies for both groups (DLD and TD), using age as the reference for normality, as described in the meta-analysis by Skoe et al.^21^
4 Before starting the time series analysis, we assessed the signals collected and exported to MATLAB (from ASCII files). In these files, three volunteers from the DLD group were registered with different parameters: stimulus intensity (70 dB instead of 80 dB), number of stimulations (sweeps), sampling period, and filtering. Therefore, the records of these volunteers were removed from the sample, as these parameters could decisively affect the results of this experiment. There remain six volunteers in the DLD group and nine volunteers in the control group (TD).
5 In the DLD group, there were only records from the left ear for six volunteers; after removing records with different collection parameters, only four remained. Due to this small number, we focused only on the analysis of the right ear records.
6 The recording of a volunteer in the control group (TD) had a signal-to-noise ratio below one. Records with a signal-to-noise ratio below 1.5 are discarded or collected again.^1^ This volunteer was removed from the sample for subsequent analyses (Fig. 1).

**Fig. 1:**
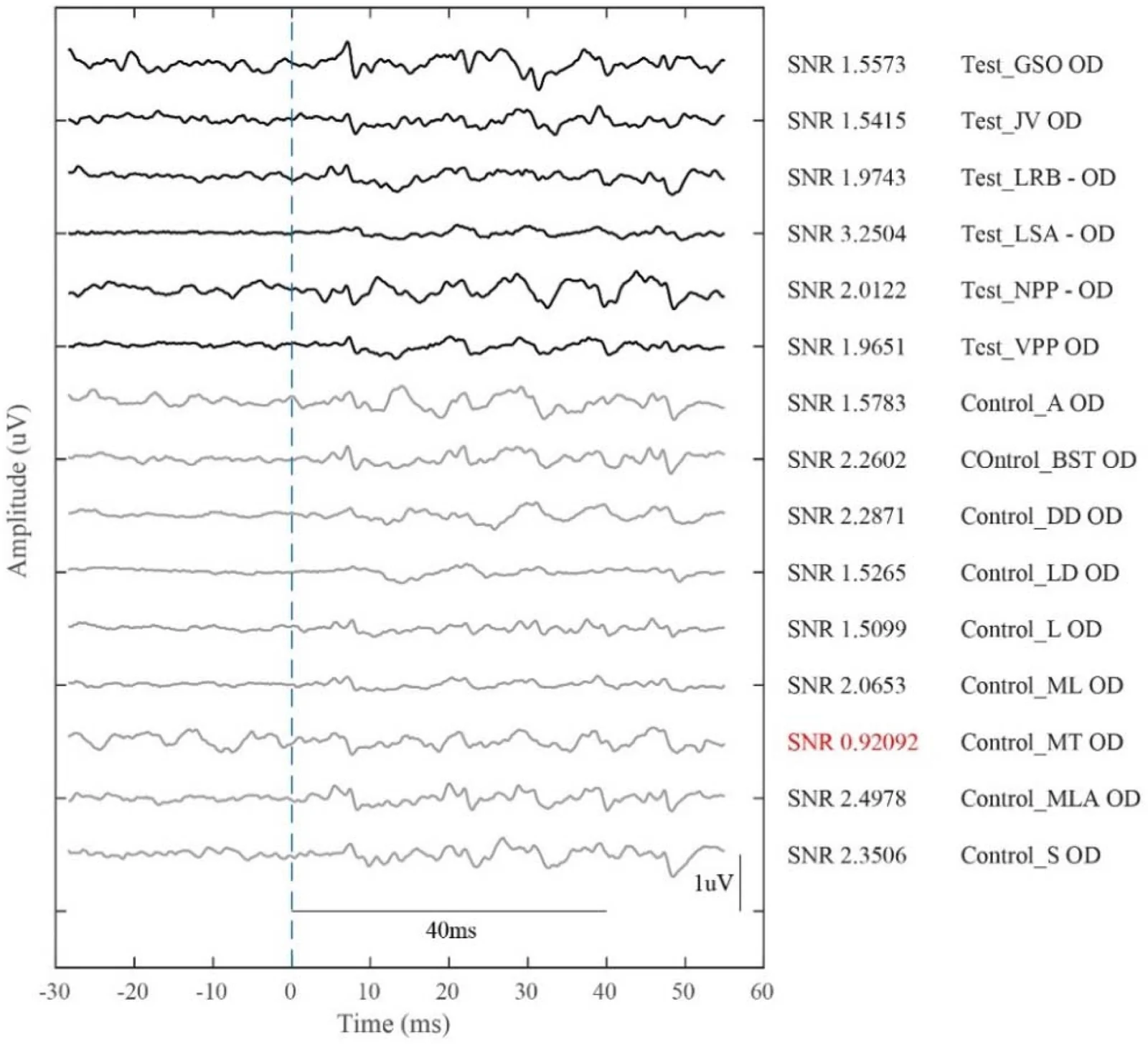
Averages of de volunteers, showing the pré and post- stimulus portions. Start of, stimulation shown by the dotted line. In red volunteer from the TD group with SNR less than 1.5 and excluded from the sample.

Therefore, the MATLAB analysis was performed only for the right ear of eight TD-group volunteers and six DLD-group volunteers.

### Statistical analysis

Children's sex was described within groups using absolute and relative frequencies, and the association between groups was assessed using Fisher's exact test. Age, latencies, and amplitudes evaluated on the right side were described within groups using mean and standard deviation and compared between groups using the unpaired Student’s *t*-test.^23^ The latency values for each age group, as reported in the literature, were summarized as means and standard deviations and compared with the respective reference using the unpaired Student's *t*-test.^23^ The assumptions of normality were assessed before the application of the parametric tests.

Statistical analyses were performed using IBM-SPSS for Windows, version 22.0, and data tabulation was performed using Microsoft Excel 2013. The tests were conducted at the 5% significance level. Post-hoc achieved power for the frequency-domain comparisons was calculated from the reported *t*-values, the final sample sizes (DLD = 6; TD = 8), and a two-sided alpha of 0.05.

## Results

Table 1 shows that there was no statistically significant difference in children's age between the groups (p = 0.279). However, there was an imbalance in sex distribution between the groups, with more male children in the DLD group, although the difference was not statistically significant (p = 0.050).

**Table 1:** Description of the children's personal characteristics according to groups and results of the statistical tests.

Variable	Typical Development (TD) (n = 9)	DLD (Developmental Language Disorder) (n = 9)	p
Age (years), mean ± SD	7.8 ± 2.4	6.4 ± 2.6	0.279
Sex, n (%)			0.050a
Female	6 (66.7)	1 (11.1)	
Male	3 (33.3)	8 (88.9)	

^a^ Unpaired Student’s *t*-test; ^a^ Fisher's Exact Test.

### Time-domain analysis

According to Table 2, there was no statistically significant difference in mean latency between the groups in the VA complex (p = 0.225), nor in any of the latencies (p > 0.05).

**Table 2:** Description of the VA complex and latencies in various waves on the right side according to groups and results of the comparative tests.

Variable	Typical Development (TD) (n = 9)	DLD (Developmental Language Disorder) (n = 9)	p
VA Complex	0.23 ± 0.10	0.31 ± 0.14	0.225
Wave V	7.32 ± 0.37	7.07 ± 0.52	0.256
Wave A	8.61 ± 0.34	8.63 ± 0.63	0.938
Wave C	13.5 ± 1.5	14.4 ± 2.3	0.330
Wave D	23.5 ± 0.6	24.2 ± 3.1	0.490
Wave E	32.5 ± 0.9	32.7 ± 1.8	0.778
Wave F	41.1 ± 1.0	40.9 ± 1.1	0.711
Wave O	48.9 ± 0.6	49.2 ± 1.2	0.580

*Unpaired Student’s *t*-test.

According to Table 2, there was no statistically significant mean difference between the groups in the VA complex (p = 0.225) nor in any of the latencies (p > 0.05).

Below are the comparison results by age group, divided into three groups: greater than 3- to 5-years, greater than 5- to 8-years, and greater than 8- to 11-years. Such groups were defined based on the meta-analysis carried out by Skoe et al.^21^

### Frequency-domain analysis

The analysis of the Signal-to-Noise Ratio (SNR) of the averages across all volunteers, calculated as the ratio of the Root Mean Square (RMS) of the signal portion of the record to the RMS of the pre-stimulus portion. The signal-to-noise ratios are shown in the figure below, along with the average evoked potential traces for each volunteer (Fig. 1).

Here, analysis in the frequency domain began. For this, Fast Fourier Transform (FFT) was applied to the sections of interest (sustained portion of the FFR). The stimulus that originally had a sampling rate of 48 kHz was resampled at 8 kHz – the same frequency as the recordings. Then, the areas of interest were determined for which the FFT was applied: in the case of the stimulus, the FFT was applied in the area of the vowel /a/, between 10 ms and 40 ms; and in evoked potentials, considering the mean latency between stimulus and response, the region of interest was defined between 15 ms and 50 ms. The results are shown in Fig. 2.

**Fig. 2:**
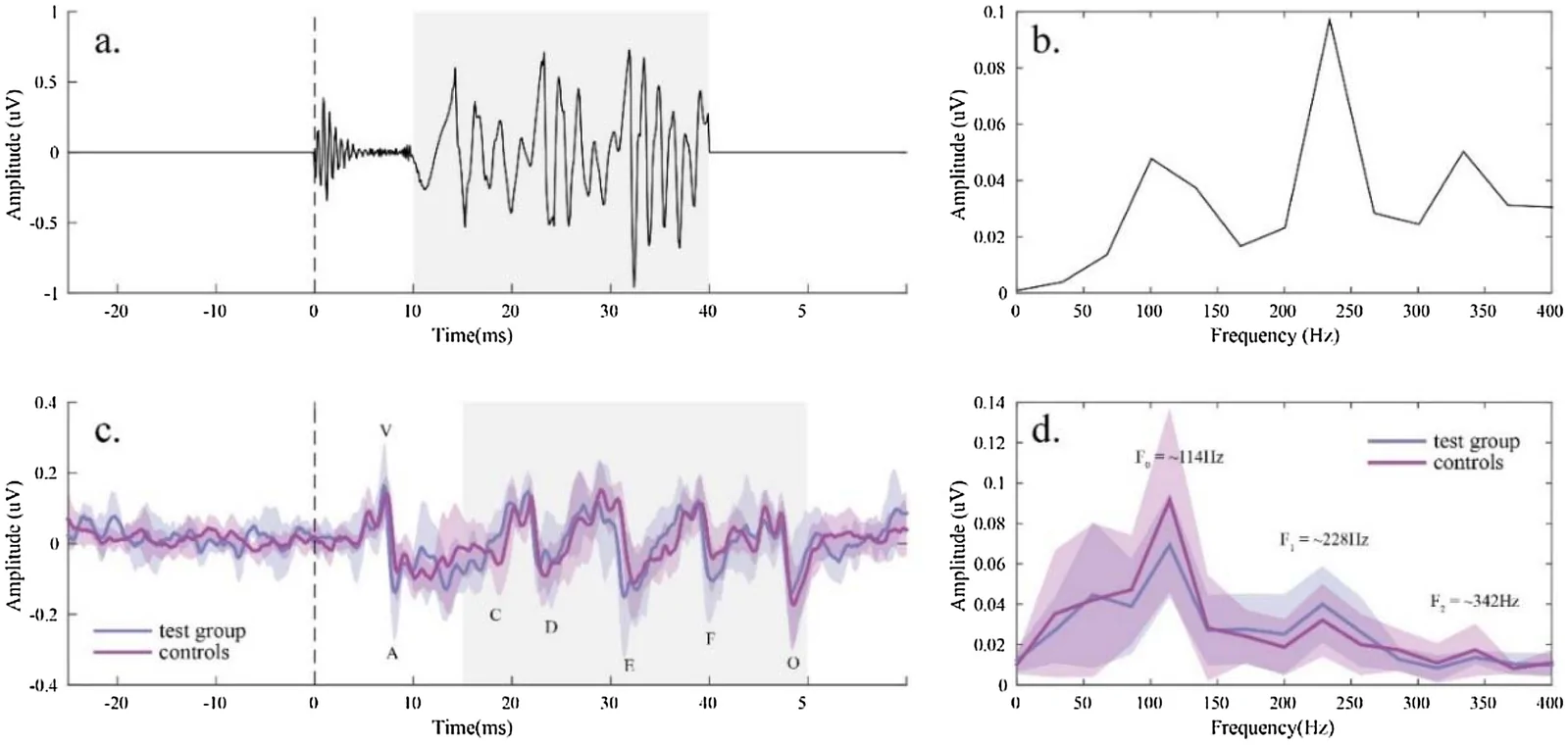
Stimuli and means of evoked potentials per group. (a) /da/ stimulus with 40 ms duration. The gray area marks the region of the vowel /a/, which was used to extract the frequencies presented in the next table; (b) Amplitudes in the frequency domain obtained through the Fast Fourier Transform (FFT); (c) Average of the evoked potentials of each group represented by the opaque lines and their respective standard deviations represented by the shaded areas of the same color. The gray area marks the evoked potential region that was used to extract the frequencies presented in the next table, between 15 ms and 50 ms; (d) Mean of amplitudes in the frequency domain obtained using FFT. The shaded areas represent the respective standard deviations.

As shown, the frequency resolution was low in our analysis due to the small window, corresponding to the sustained portion of the auditory potential (which yielded 280 points). However, this did not affect our analyses, as the stimulus did not maintain a sustained static frequency for long, leading the responses to vary between 103 and 125 Hz, as reported in other studies in the specialized literature using this same stimulus.^24^ In any case, a window would be used to compare the amplitude in the frequency bands of interest. Therefore, other frequency-domain analyses would not offer an advantage over the Fast Fourier Transform applied here.

Afterwards, the amplitudes of each volunteer were quantified in the frequency bands of interest: f0 = ∼114 Hz, f1 = ∼228 Hz, and f2 = ∼342 Hz.

When comparing amplitudes between groups using the t-test, no statistically significant results were observed for f0 [t(12) = 1.17, p = 0.27], f1 [t(12) = -0.79, p = 0.45], or f2 [t(12) = 0.80, p = 0.44] (Fig. 3). Post-hoc power was low for these comparisons (0.190, 0.113, and 0.114, respectively), indicating that the results should be interpreted descriptively.

**Fig. 3:**
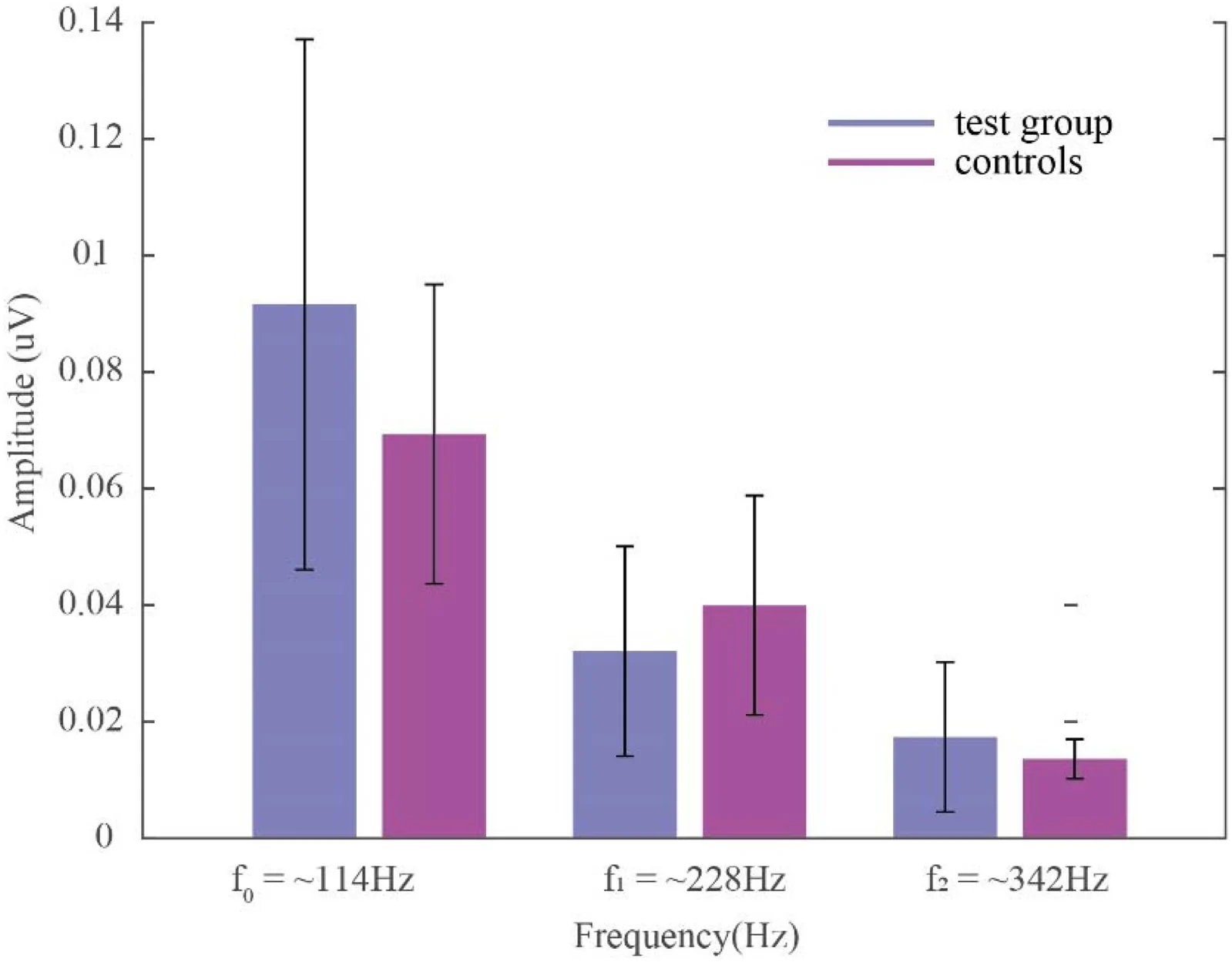
Bar graph with means and standard deviation of the amplitudes of the frequencies of interest for both investigated groups.

## Discussion

Children with DLD are known to have difficulty perceiving speech, and this condition can be aggravated in the presence of noise. Noise degrades acoustic input and requires more robust linguistic knowledge, which is already impaired in these children. In the presence of noise, children with DLD need to combine auditory input and linguistic memory representations from partial information, which can impact normal language development and acquisition.^25^ These difficulties will accompany the child throughout its development and, if left undiagnosed and untreated, will lead to difficulties in school and interpersonal interactions.

Studies link DLD difficulties to auditory-perceptual problems, specifically speech sounds' temporal processing. Deficits in perceiving speech's rapidly changing temporal (milliseconds) and spectral characteristics cause unstable or inaccurate phoneme representations in the brain. These unstable representations can impair speech perception and language-learning abilities in phonology, syntax, or semantics.^25^

The perception of speech begins in the auditory brainstem and is closely related to language acquisition and learning. The FFR is an objective way to investigate this process and allows the identification of auditory processing deficits associated with communication skills in a context similar to everyday dialogue.^26^

The present study analyzed and compared FFR in children with Developmental Language Disorder and in a control group with typical development. Given that many of these children in the clinical case group (DLD) may exhibit alterations in the temporal processing of speech sounds, it would be possible to objectively assess this difficulty in the temporal and frequency domains of the FFR.

In the initial time-domain analysis of the amplitude of the VA complex and absolute latencies of waves V, A, C, D, E, F, O, in the case group and in the control group, for the right ear collected in the study (Table 2), there was no statistical significance when comparing wave latencies between case and control groups.

Comparison between age groups, grouped according to the meta-analysis by Skoe & Kraus,^21^ according to Table 3, in all evaluated age groups, the latency values for both the Typical Development (TD) group and the DLD (Developmental Language Disorder) group were, on average, statistically higher than those in reference literature (p < 0.05).

**Table 3:** Description of latencies in the different age groups and study groups, and results of the comparisons with their respective reference values.

Variable/Age group	Reference (mean ± SD)	Typical Development (TD) (mean ± SD)	p	DLD (Developmental Language Disorder) (mean ± SD)	p
Wave V					
3–5 years	6.59 ± 0.26	7.38 ± 0.35	<0.001	7.31 ± 0.63	<0.001
6–8 years	6.45 ± 0.21	7.50 ± 0.13	<0.001	6.73 ± 0.46	0.031
9–11 years	6.45 ± 0.25	7.16 ± 0.49	<0.001	7.10 ± 0.14	<0.001
Wave A					
3–5 years	7.56 ± 0.35	8.63 ± 0.35	<0.001	8.69 ± 0.80	<0.001
6–8 years	7.39 ± 0.28	8.67 ± 0.38	<0.001	8.45 ± 0.64	<0.001
9–11 years	7.37 ± 0.36	8.57 ± 0.42	<0.001	8.79 ± 0.58	<0.001
Wave D					
3–5 years	22.36 ± 0.38	23.57 ± 1.32	<0.001	23.32 ± 1.11	<0.001
6–8 years	22.16 ± 0.28	23.67 ± 0.56	<0.001	25.73 ± 5.44	<0.001
9–11 years	22.27 ± 0.56	23.35 ± 0.41	<0.001	23.84 ± 1.36	<0.001
Wave E					
3–5 years	30.90 ± 0.37	32.57 ± 0.62	<0.001	32.16 ± 1.12	<0.001
6–8 years	30.82 ± 0.45	32.13 ± 0.45	<0.001	33.73 ± 2.67	<0.001
9–11 years	30.79 ± 0.44	32.85 ± 1.25	<0.001	32.39 ± 1.43	<0.001
Wave F					
3–5 years	39.34 ± 0.32	42.19 ± 1.68	<0.001	40.97 ± 0.95	<0.001
6–8 years	39.23 ± 0.27	40.38 ± 0.22	<0.001	41.33 ± 1.71	<0.001
9–11 years	39.34 ± 0.52	41.16 ± 0.67	0.000	40.29 ± 0.13	0.007
Wave O					
3–5 years	48.14 ± 0.42	48.75 ± 0.71	0.026	49.53 ± 1.68	<0.001
6–8 years	47.88 ± 0.31	48.67 ± 0.29	<0.001	49.03 ± 0.84	<0.001
9–11 years	48.10 ± 0.48	49.22 ± 0.70	<0.001	48.69 ± 0.44	0.048

*Unpaired Student’s *t*-test.

In MATLAB's frequency-domain FFR analysis using Fast Fourier Transform (FFT), the absence of statistically significant group differences should be interpreted cautiously in light of the limited post-hoc power, rather than as evidence of equivalence. The processing pipeline nevertheless represents a methodological contribution, because it applied an explicit spectral approach to the sustained FFR after export from SmartEP to MATLAB. The large wave-summation averages (6,000 sweeps) showed wave latencies, f0, and formant-related bands consistent with specialized literature and tutorials by Skoe & Kraus,^1^ Krizman & Kraus,^22^ and Anderson & Kraus,^24^ supporting reproducibility under established parameters.

Elmahallawi TH et al.^25^ studied FFR in noise in children with DLD to investigate speech processing in silence and noise. The study included a control group (15 typically developing children) and a study group (25 children with DLD), aged 3–6 years. Children underwent intelligence, language, and audiological assessments, plus FFR in silence and noise. In silence, no significant differences emerged between ears or groups in Fundamental Frequency and first Formants amplitudes. A larger sample and a case group with marked temporal processing alterations are likely necessary.

Barman et al.^27^ evaluated subcortical coding in ten children aged 4 to 7-years, comparing five children with DLD and normal bilateral hearing with five typically developing controls.

Waveforms obtained from Typically Developing (TD) children were clearer and more easily identifiable, with larger negative peaks observed. A sharper and smoother VA complex was observed (in the TD group). Waveform morphology was relatively worse in most children with DLD. Shallower peaks, particularly shallower VA slopes, were seen in the DLD group.^27^ This can be observed in our study, where the DLD group also showed waves with worse morphology and greater difficulty in marking (by two audiologists experienced in electrophysiology), as well as a clearer VA complex with greater amplitudes in the control group.

Mean latency in the DLD group was prolonged compared to TD children, suggesting delayed information transmission to higher centers. Waves “C” and “D” latencies were significantly delayed bilaterally in children with DLD, while wave “V” latency showed no significant inter-ear difference. FFR component amplitudes also showed no significant inter-group differences bilaterally.^27^ Despite our larger sample size, no significant absolute wave latency differences emerged between case and control groups.

The FFR can be a promising test for the follow-up of these children and for therapeutic aid, as it enables understanding of the physiological bases of these disorders. It also allows the evaluation of the temporal processing of speech sounds in younger children.

Throughout the collection process, tracing remained stable in spontaneous sleep or wakefulness, with the child sitting or lying down, yielding reliable, replicable results.

The absence of statistical significance for time- and frequency-domain parameters is therefore best understood as a limitation of statistical power, rather than evidence of absence of group differences. DLD heterogeneity was acknowledged; however, all children in the DLD group shared phonological impairment plus at least one additional altered language subsystem, supporting the exploratory grouping under a phonological/temporal speech-processing hypothesis while still limiting generalizability. The borderline imbalance in sex distribution between the groups (p = 0.050), with a higher proportion of male participants in the DLD group, may represent a potential confounding factor and should be considered a limitation of this study. Because this exploratory pilot study focused on between-group comparisons, no formal correlation analysis was performed among the time- and frequency-domain parameters. Therefore, the relationships among these measures should be interpreted cautiously.

## Conclusion

In this exploratory pilot study, FFR acquisition and time- and frequency-domain analyses were feasible in children with Developmental Language Disorder and typically developing children. No statistically significant differences were observed between groups in the analyzed FFR parameters, likely reflecting the small sample size and clinical heterogeneity. These findings suggest that FFR may represent a useful complementary objective tool for investigating speech-sound encoding in children with DLD, while larger studies are needed to clarify its clinical and neurophysiological relevance.

## ORCID ID

Doris Ruthy Lewis: 0000-0002-7110-147X

## Declaration of Generative AI and AI-assisted technologies in the writing process

During the preparation of this work the author(s) used Manus 1.6 in order to format the original article according to BJORL publication guidelines. After using this tool/service, the author(s) reviewed and edited the content as needed and take/ took full responsibility for the content of the published article.

## Funding

This work was supported by the Coordenação de Aperfeiçoamento de Pessoal de Nível Superior ‒ Brasil (CAPES) [Modality 2 scholarship awarded to Gustavo Fernando Tognini Rodrigues].

## Data availability statement

The authors declare that all data are available in repository.

## Meeting information

Not applicable.

## Declaration of competing interest

The authors declare no conflicts of interest.
